# Supplementary material for: Synergistic growth of nickel and platinum nanoparticles via exsolution and surface reaction
Source: Nat Commun. 2024 May 13;15:4007. doi: 10.1038/s41467-024-48455-2 (PMC11091132; doi:10.1038/s41467-024-48455-2)
Supplement: Supplementary file 1 — Supplementary Information [file 41467_2024_48455_MOESM1_ESM.pdf]

Supplementary Information

Synergistic Growth of Nickel and Platinum Nanoparticles via Exsolution and Surface Reaction

Min Xu[1,†], Yukwon Jeon[2,†], Aaron Naden[1], Heesu Kim[2], Gwilherm Kerherve[3], David J. Payne[3,4], Yong-gun Shul[5] and John T.S. Irvine[1*]

*Correspondence to: jtsi@st-andrews.ac.uk (Prof. John T.S. Irvine).
†These authors contributed equally to this work.

This PDF file includes:

Figures S1 to S21
Tables S1 to S5
References (1-6)

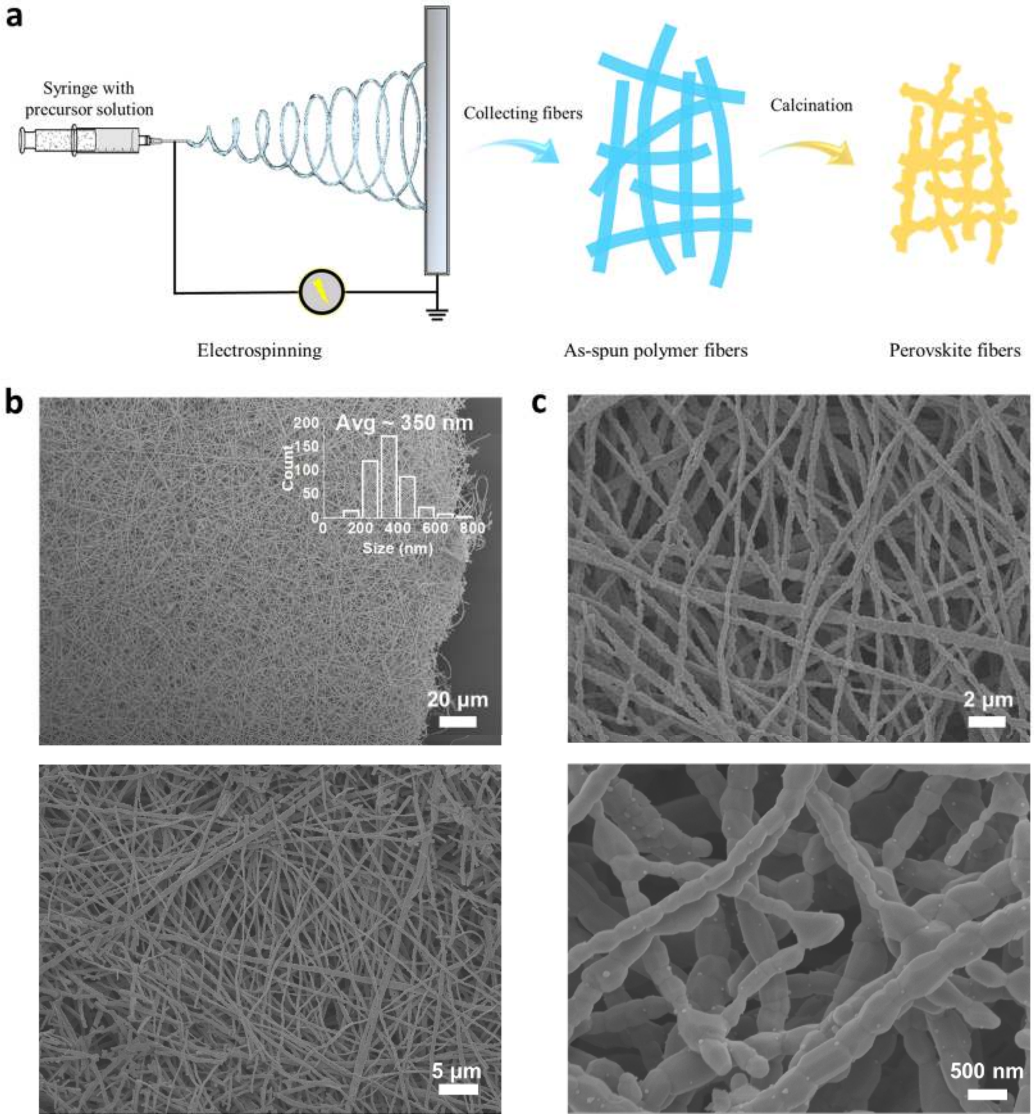

Supplementary Figure 1. (a) Schematic of the preparation procedure for perovskite nanofibers. The as-spun nanofibers are prepared by electrospinning and collected from the collector, then dry at 80 ºC for 5h followed by calcined at 1100 ºC for 2h. SEM images for the (b) LCNT nanofibrous web and (c) LCNT reduced nanofibers at 800 ºC for 4h in 5% H_2/Ar.

The prepared perovskite nanofibrous nonwoven matrix in Fig.1 and **Supplementary Figure** 1 is considered as a support due to their high aspect ratio that allows appropriate 3D open-configuration, providing relatively high surface area, easily accessible pores for better mass diffusion and prohibiting the coalescence during a long operating period by less contact points[1].

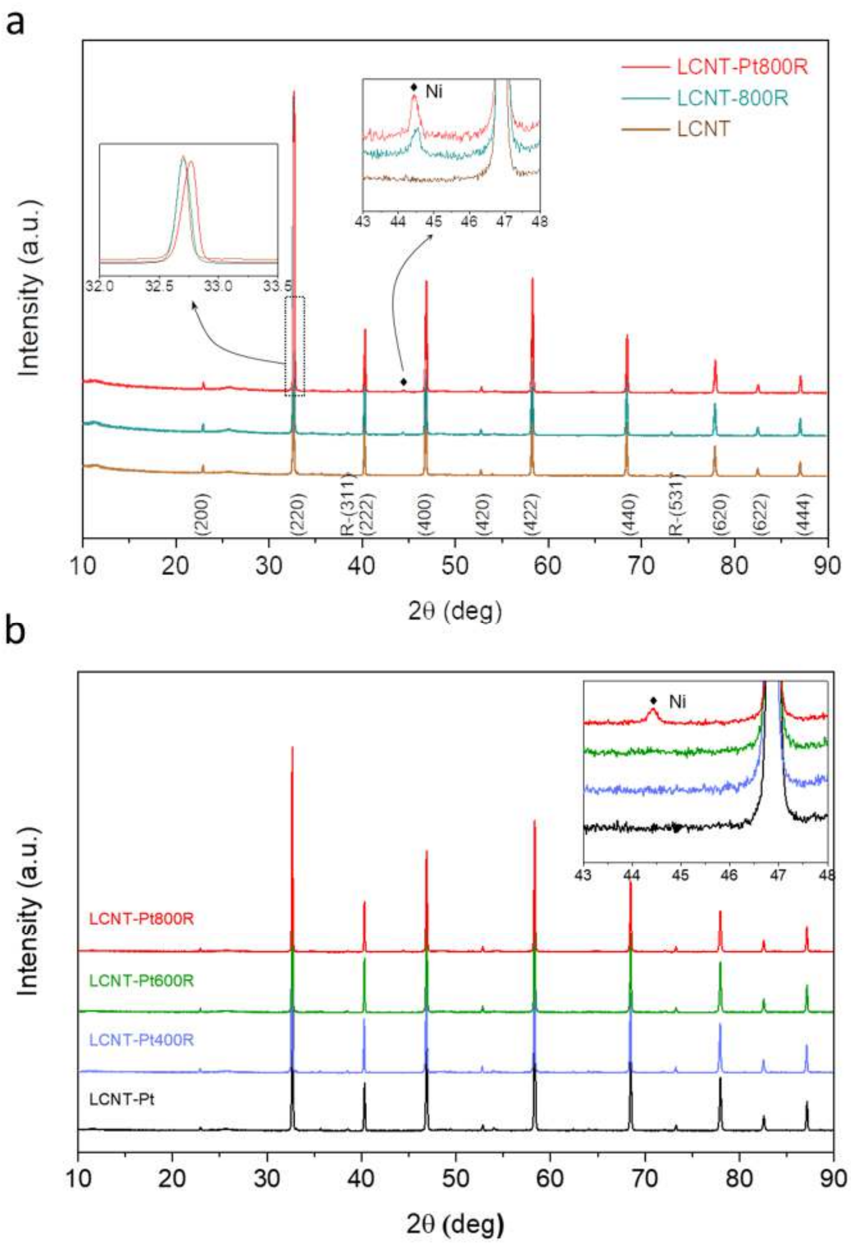

Supplementary Figure 2. XRD patterns of (a) $La_{0.52}Ca_{0.28}Ni_{0.06}Ti_{0.96}O_3$ fiber calcined at 1100 °C in air and $La_{0.52}Ca_{0.28}Ni_{0.06}Ti_{0.96}O_3$ fiber reduced at 800 °C for 4 h in 5% H_2/Ar, inset: magnification for 32 ° – 33.5 ° corresponding to the main peak, and 43 °- 48 ° corresponding to the metallic Ni or NiPt.
(b) Pt- $La_{0.52}Ca_{0.28}Ni_{0.06}Ti_{0.94}O_3$ nanofibers calcined at 400 °C for 2 h in air and following with reduction at 400 °C, 600 °C, 800 °C for 4 h in 5% H_2/Ar. (Inset: magnification for 43°-48° corresponding to metallic NiPt).

From the X-ray diffraction (XRD) analysis Fig.S2, pristine LCNT fibers before reduced revealed sharp peaks corresponding to crystalline single phase with tetragonal structure and no metallic Ni. XRD patterns show obvious peak at 2θ positions of 44° assigned to metallic Ni correspond to (111) reflections.

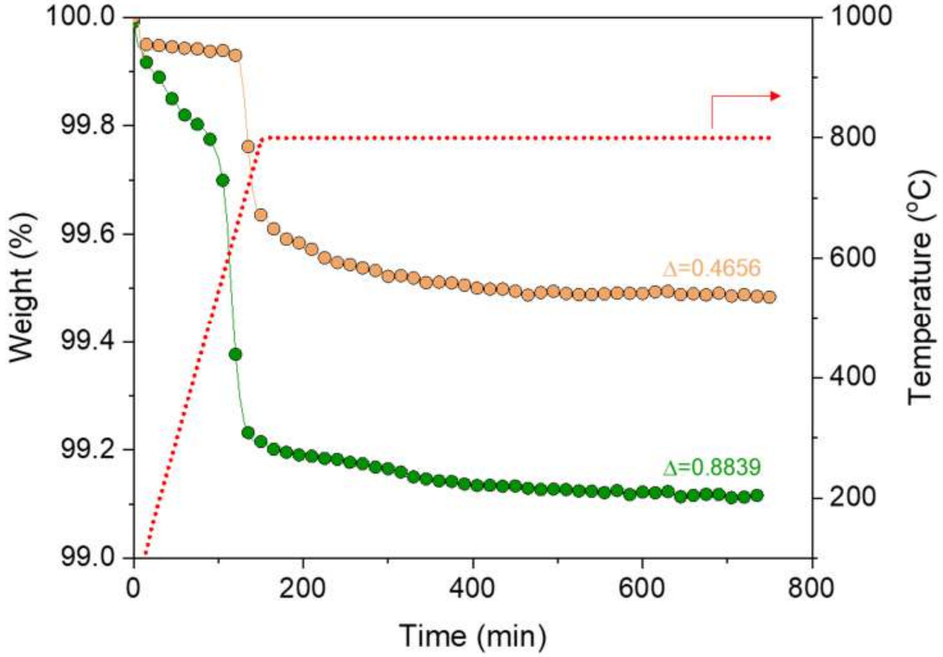

Supplementary Figure 3. TGA plot of weight loss as a function of reduction time for LCNT and LCNT-Pt nanofibers. The data was collected from room temperature to 800 °C and then has a 10 h isothermal step, under 20 mL/min, 5 % H_2/Ar flow. The inset value shows a weight loss after isothermal procedure. Based on the change in mass, we can calculate the corresponding formation of oxygen vacancies. Considering the reduction of a perovskite ABO_3 to $ABO_{3-\delta}$, δ is the oxygen deficiency.

Lattice oxygen is stripped from perovskite upon heating in reducing atmosphere while one oxygen atom taking out accompanied with an oxygen vacancy and two electrons formed:

$$\mathrm{O_O^x} \rightarrow \frac{1}{2} O_2 + \mathrm{V_O^{\cdot\cdot}} + 2e'$$

Considering about the conserved mole number and corresponding mass change, an expression based on molar weights ($M_{ABO_3}$and $M_{ABO_{3-\delta}}$) equality is given as following:

$$\frac{M_{ABO_3}}{M_{ABO_{3-\delta}}} = \frac{m_{ABO_3}}{m_{ABO_{3-\delta}}}$$

When oxygen vacancy formed, this equation can be arranged as:

$$\frac{M_{ABO_3}}{M_{ABO_3} - \delta \cdot M_O} = \frac{m_{ABO_3}}{m_{ABO_{3-\delta}}} \text{ and } \frac{\delta \cdot M_O}{M_{ABO_3}} = \frac{m_{ABO_3} - m_{ABO_{3-\delta}}}{m_{ABO_3}}$$

Thus, the oxygen vacancy can be expressed as:

$$\delta = \frac{M_{ABO_3}}{M_O} \cdot \frac{m_{ABO_3} - m_{ABO_{3-\delta}}}{m_{ABO_3}}$$

The TGA data reveals that mass change of 0.76% for LCNT fiber while 1.02 % weight loss for LCNT-Pt sample. Considering about 0.5 wt% PtO of oxygen loss 0.0379 % during reduction, thus, the LCNT fiber forming a 0.085 oxygen deficiency and the LCNT-Pt fiber reaching 0.111 oxygen deficiency.

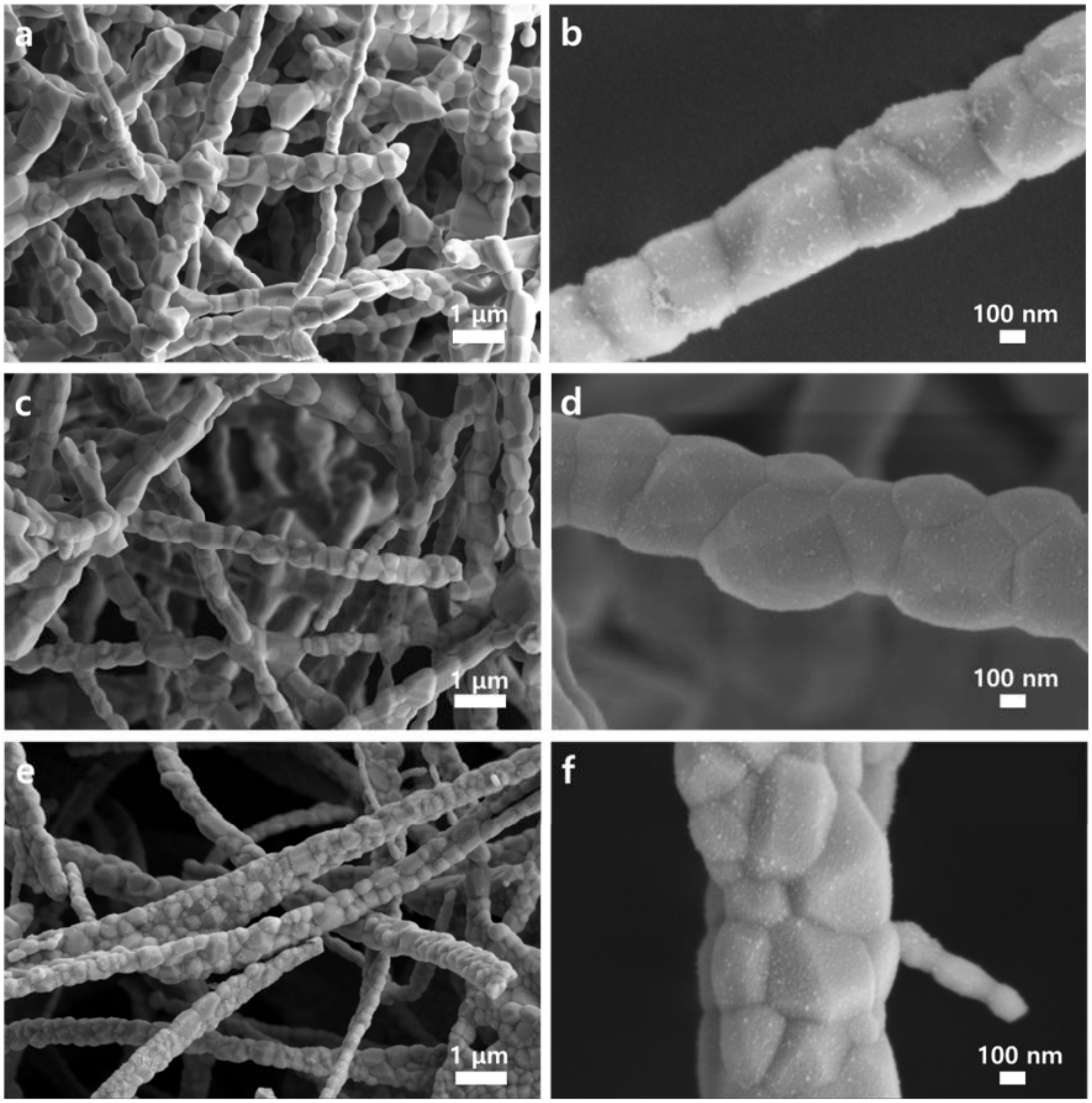

Supplementary Figure 4. SEM images for LCNT-Pt nanofibers reduced at (a-b) 400 ºC, (c-d) 600 ºC and (e-f) 800 ºC for 4 h in 5% H_2/Ar. All treated samples showing only changes in particle sizes and distributions.

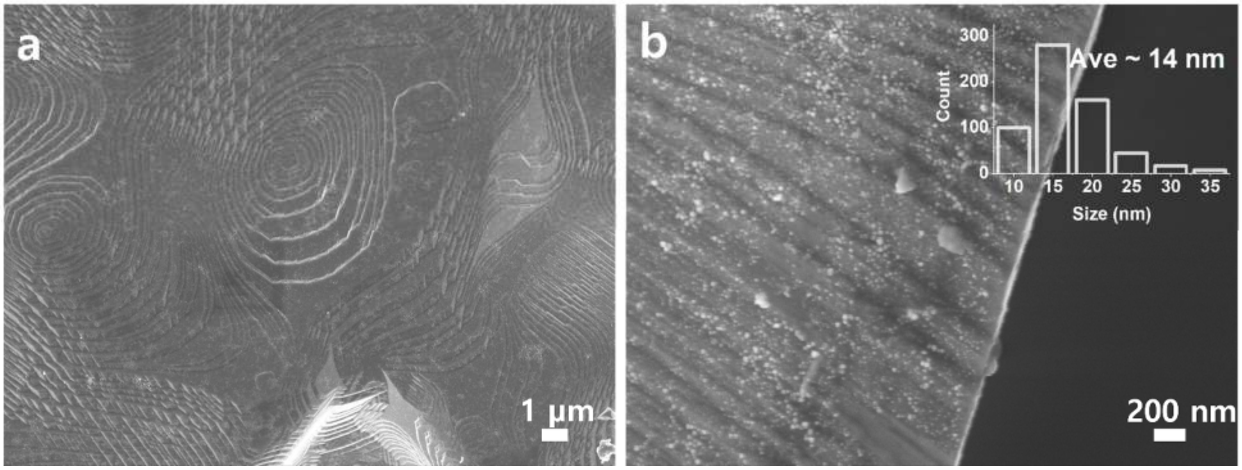

Supplementary Figure 5. SEM images of Pt nanoparticle deposition on (a-b) LCNT pellet after reduced at 800 ºC for 4 h in 5% H_2/Ar.

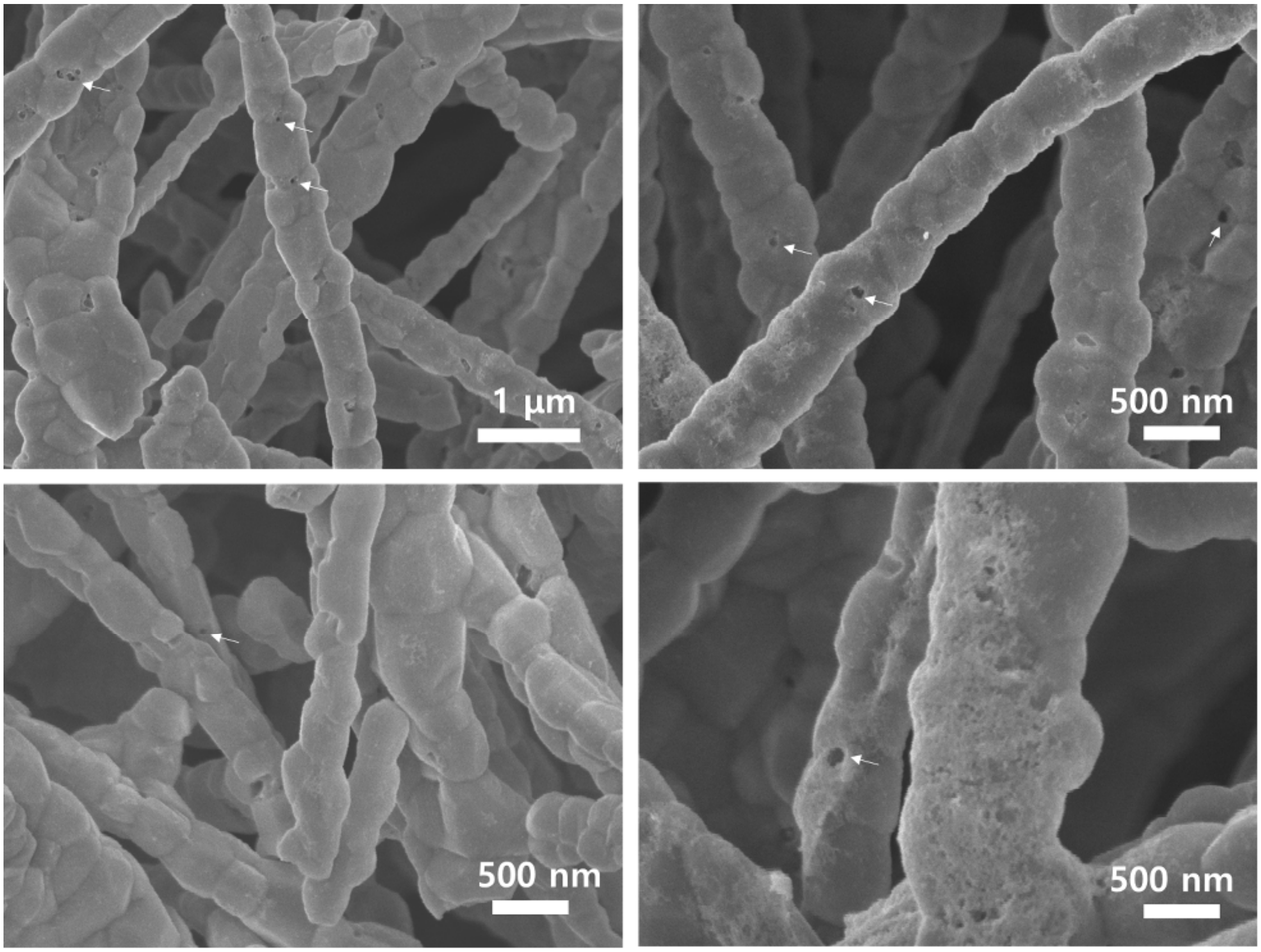

Supplementary Figure 6. SEM images of Pt nanoparticle deposition on reduced LCNT nanofibers at 800 ºC for 4 h in 5% H_2/Ar at different position. The white arrows point to the holes formed by etching of Pt nitrates precursor.

After calcination, no distinct Ni particles can be seen on the fiber surface while holes were formed from etching the exsolved Ni particles by nitric acid in our precursor solution and some pits of Pt species with uneven dispersion were observed. The perovskite titanate is resistant to diluted nitric acid in our precursor solution, with only exsolved Ni metal particles etched. Moreover, an uneven Pt dispersion on the reduced fibers can be seen with piece of Pt coated on some areas rather than fine dispersed nanoparticles indicates.

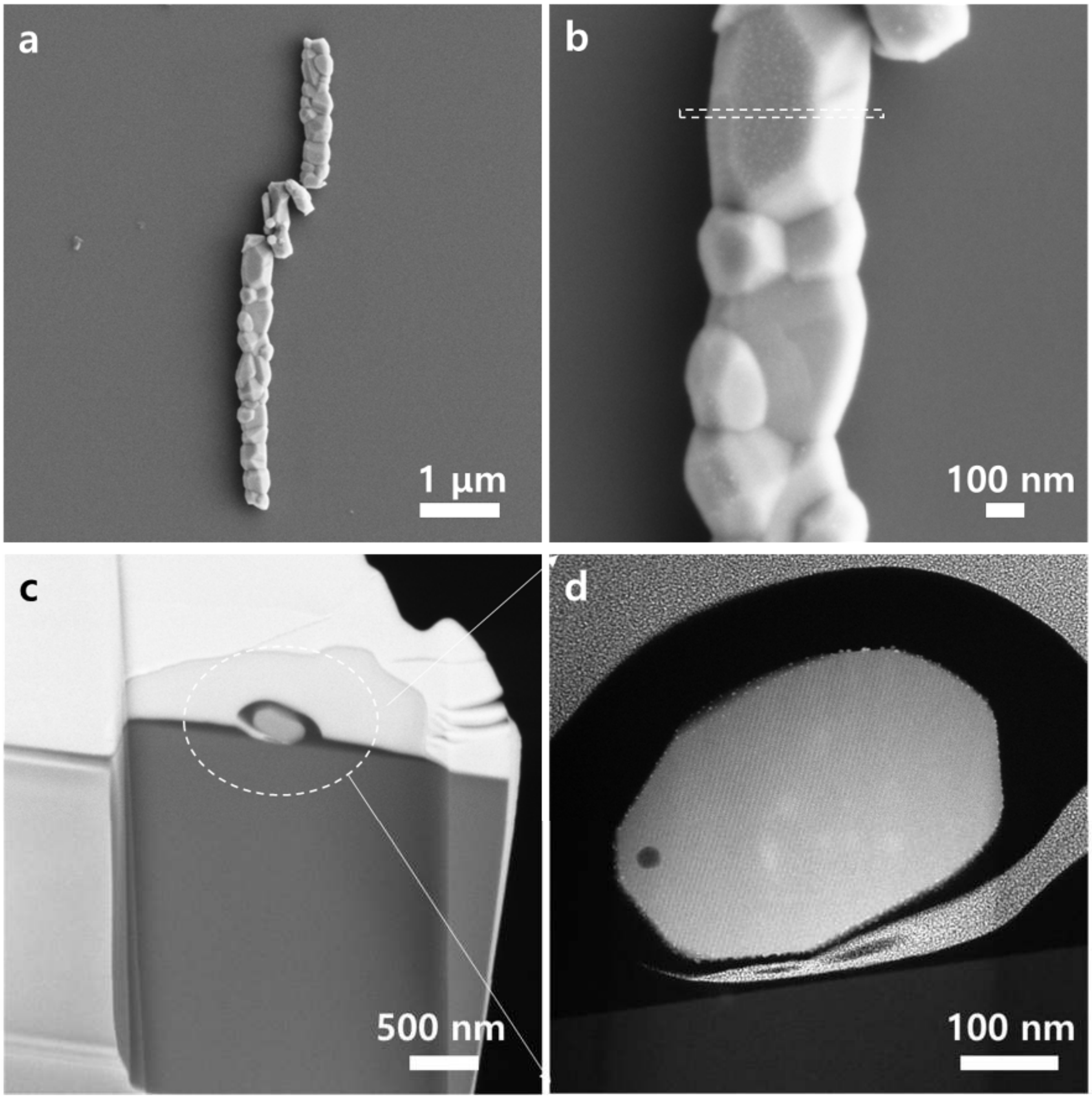

Supplementary Figure 7. Flow chart of TEM fibre sample prepared by FIB on Scios Dual-beam platform. (a) The fiber on silicon wafer piece, (b) the FIB sampled region, (c) the prepared sample for TEM, (d) TEM for the fiber cross section region.

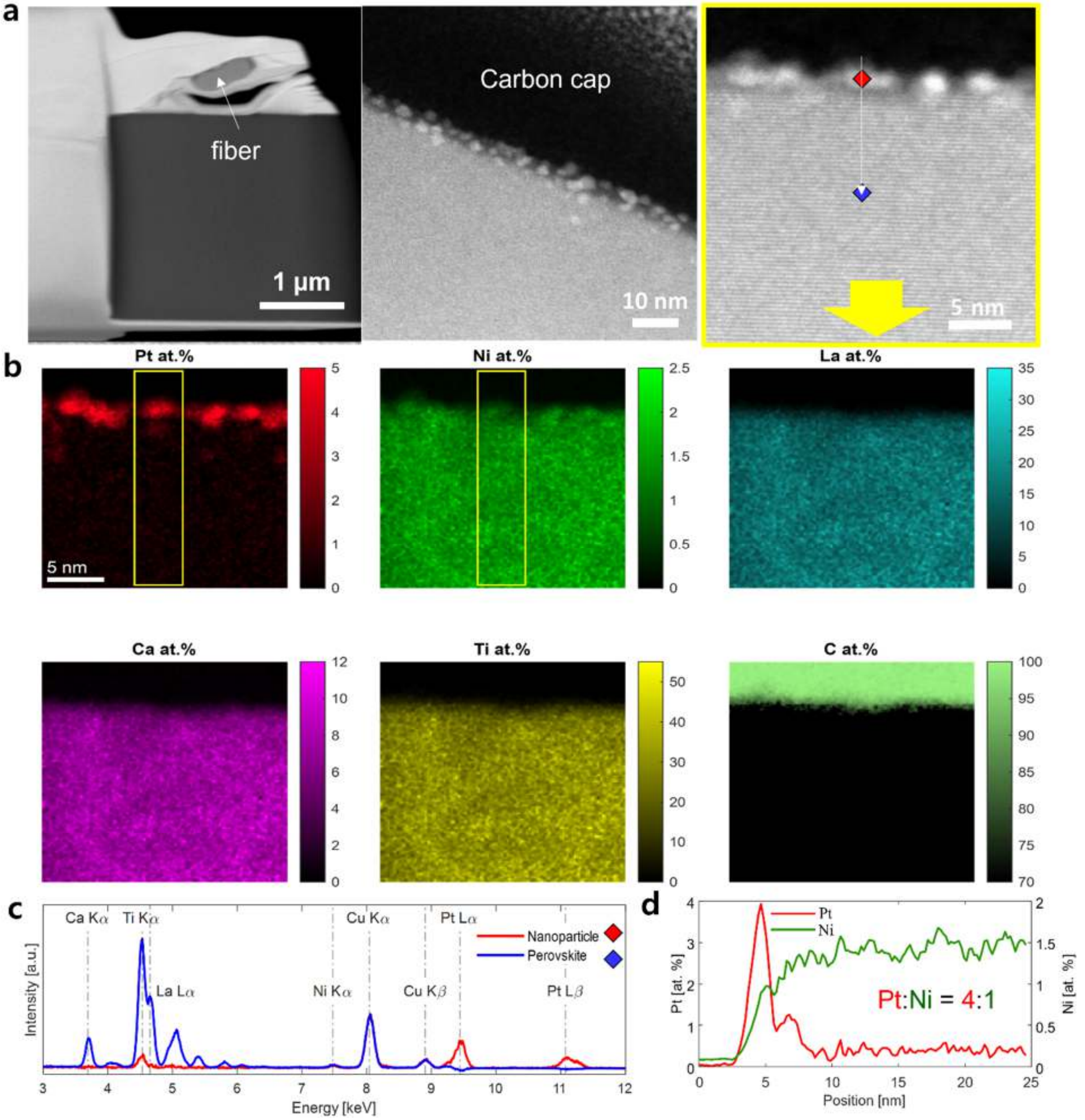

Supplementary Figure 8. Representative HAADF-STEM images and EDX analysis of LCNT-Pt nanofiber catalyst reduced at 600 ºC for 4 h in 5% H_2/Ar. The specimens was prepared by Focused Ion Beam (FIB). (a) The cross section of prepared nanofiber material and the supported nanoparticles on the perovskite surface, which are mainly wuff shape. (b) EDX mapping and (c) spot analysis with its atomic values for Pt, Ni, La, Ca, Ti, C, as well as (d) line–scan profiles analysis with its atomic values for Pt and Ni. It is clearly seen that the region around particle present less Ni than the back and even the Pt particle. The Ni to Ti ratio of the bulk is about 0.05:1, close to the design stoichiometry for the pristine fibers.

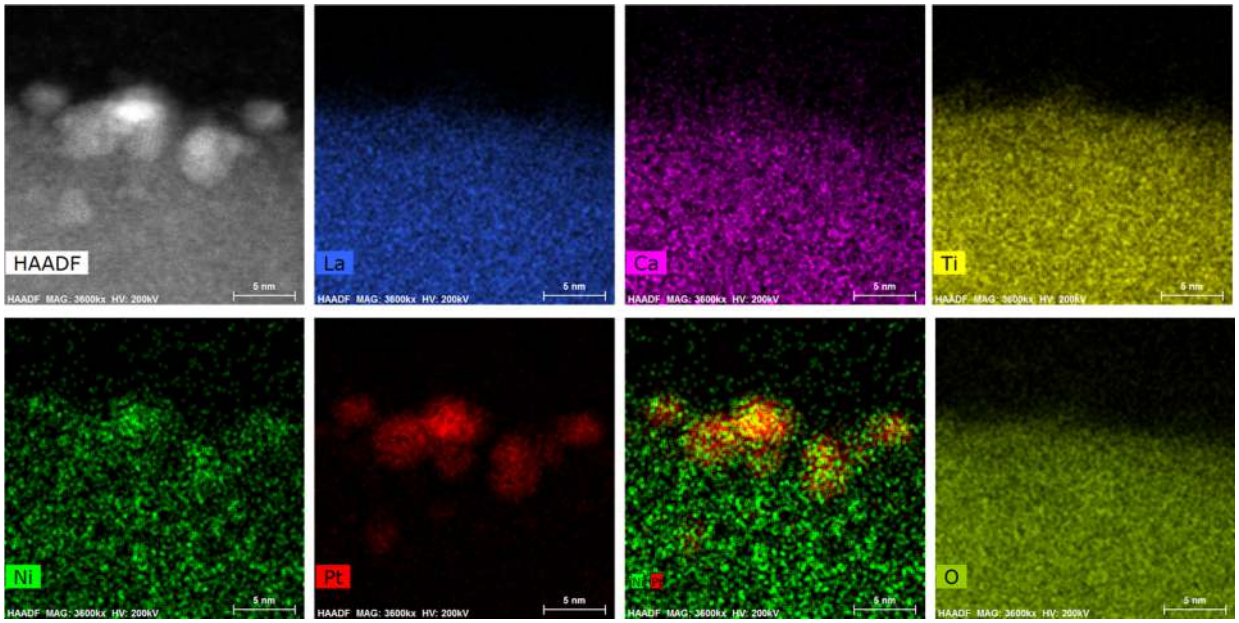

Supplementary Figure 9. Representative HAADF-STEM images and EDX analysis of LCNT-Pt nanofiber catalyst reduced at 600 ºC for 4 h in 5% H_2/Ar.

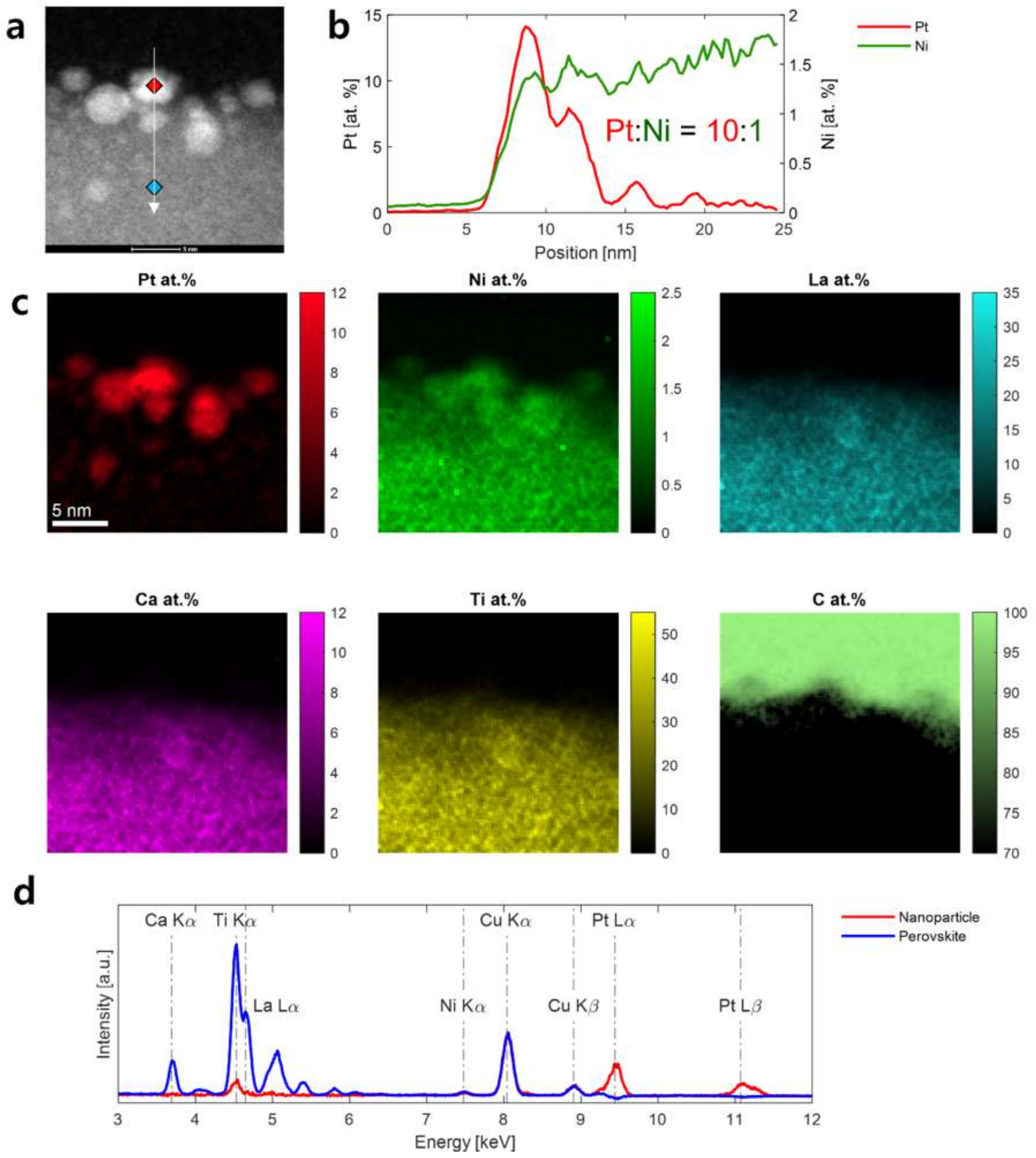

Supplementary Figure 10. Representative HAADF-STEM images and EDX analysis of LCNT-Pt nanofiber catalyst reduced at 600 ºC for 4 h in 5% H_2/Ar. The specimens was prepared by Focused Ion Beam (FIB). (a) The cross section of prepared nanofiber material and the supported nanoparticles on the perovskite surface. (b) line–scan profiles analysis with its atomic values for Pt and Ni, (c) EDX mapping, (d) the spot analysis of the particle and the bulk. It is clearly seen that the region around particle present less Ni than the back and even the Pt particle.

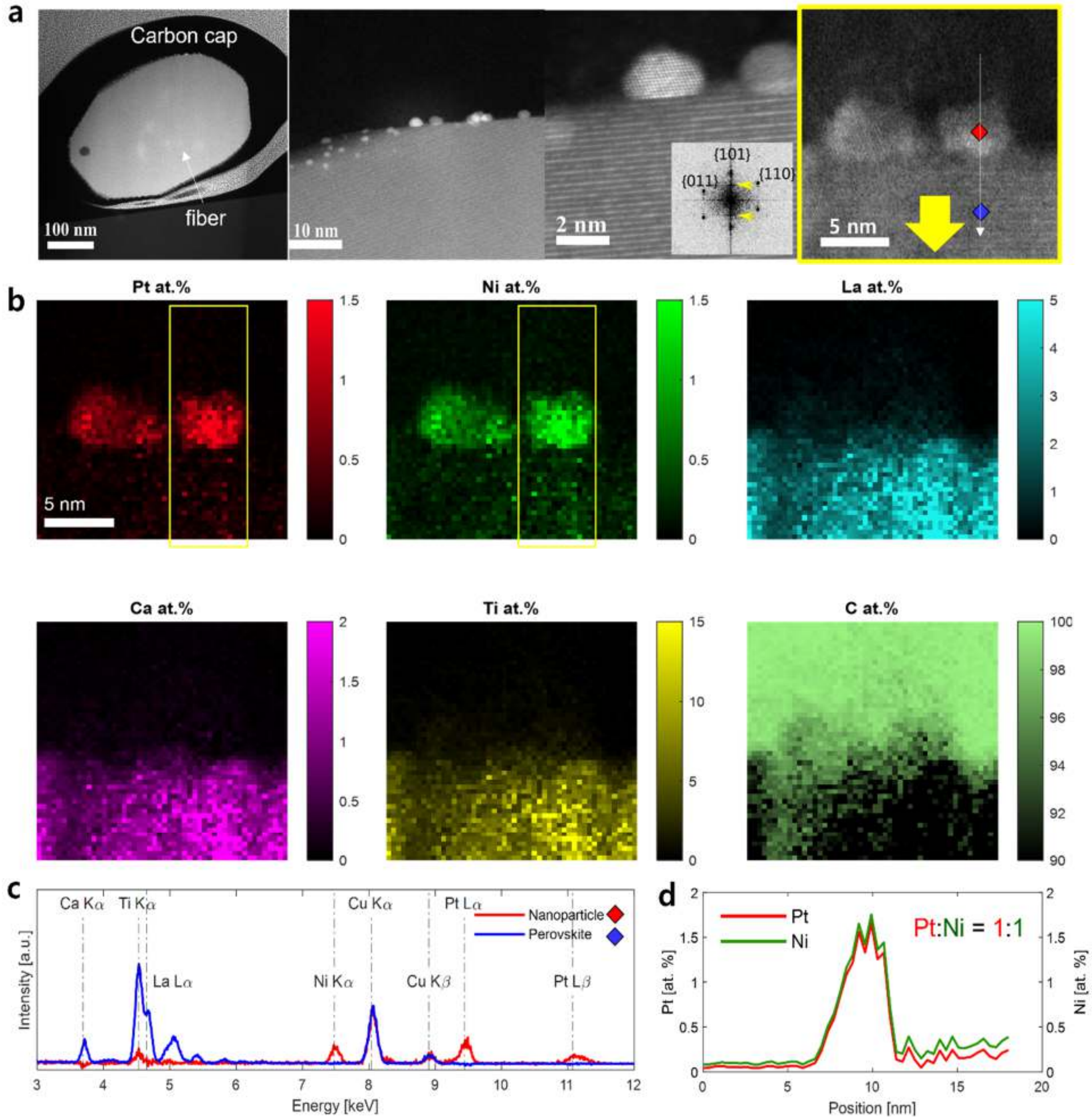

Supplementary Figure 11. Representative HAADF-STEM images and EDX analysis of LCNT-Pt nanofiber catalyst reduced at 800 ºC for 4 h in 5% H_2/Ar. The specimens was prepared by Focused Ion Beam (FIB). (a) The cross section of prepared nanofiber material and the supported nanoparticles on the perovskite surface, which are mainly wuff shape. (b) EDX mapping and (c) spot analysis with its atomic values for Pt, Ni, La, Ca, Ti, C, as well as (d) line–scan profiles analysis with its atomic values for Pt and Ni. The line profile analysis shows 1:1 atmoic ratio of Pt and Ni for the insituly Ni decorated Pt nanoparticles on the perovskite nanofibers.

The particles grow in epitaxial on (110) native surface facet of the nanofiber. This keep the growth of nanoparticle in a smooth and continuous way on the surface of fibers. The perovskite fiber retained a pseudo-cubic structure even after long term reduction.

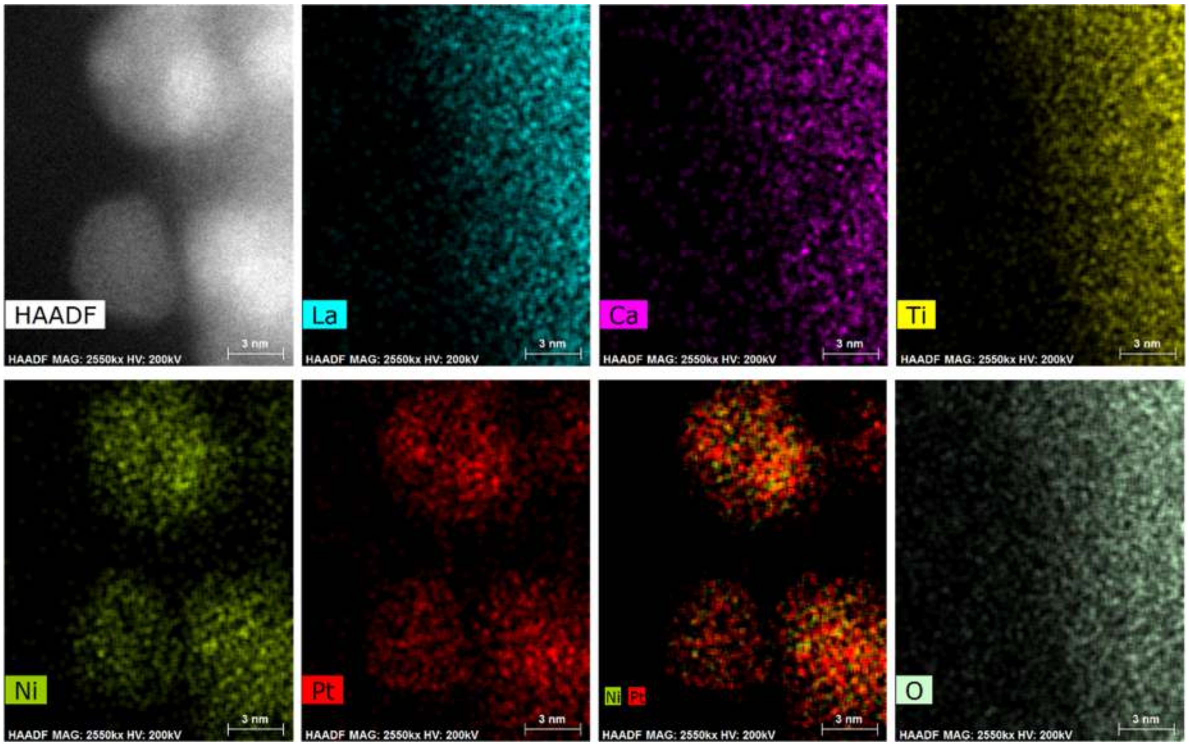

Supplementary Figure 12. Representative HAADF-STEM images and EDX analysis of LCNT-Pt nanofiber catalyst reduced at 800 ºC for 4 h in 5% H_2/Ar.

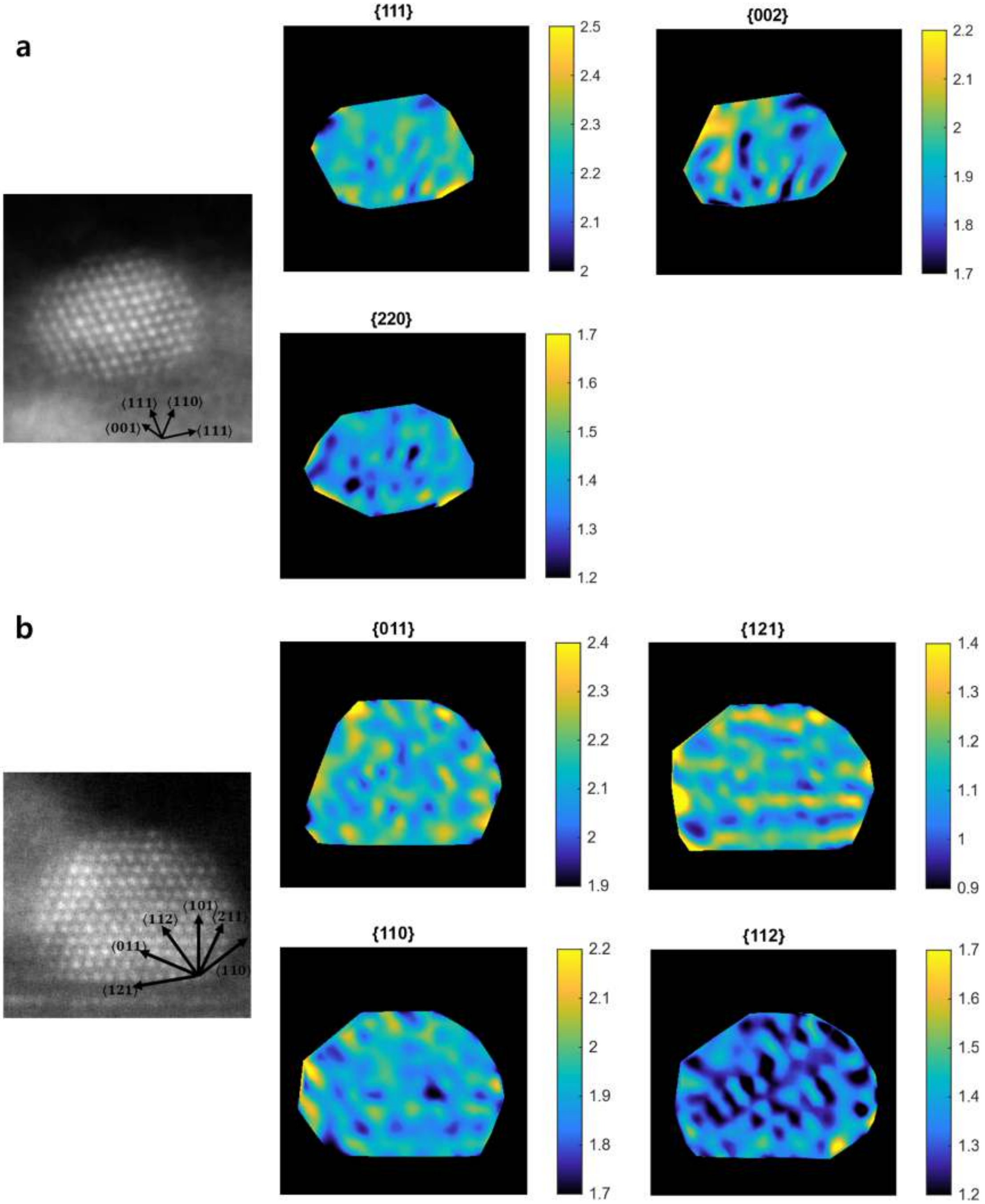

Supplementary Figure 13. Representative HAADF-STEM images and atomic resolution strain map within different crystallographic planes for the nanoparticles on LCNT-Pt nanofibers reduced at (a) 600 ºC and (b) 800 ºC for 4 h in 5% H_2/Ar. The bimetallic particles are epitaxial growth on the support with a truncated Wulff-shape.

It is worth to note that the Wulff shape is mainly for an isotropic medium, thus revealed thermodynamically stable configuration[2] with uniform dispersion of the single-phase Pt-Ni alloy nanoparticles for LCNT-Pt 800R.

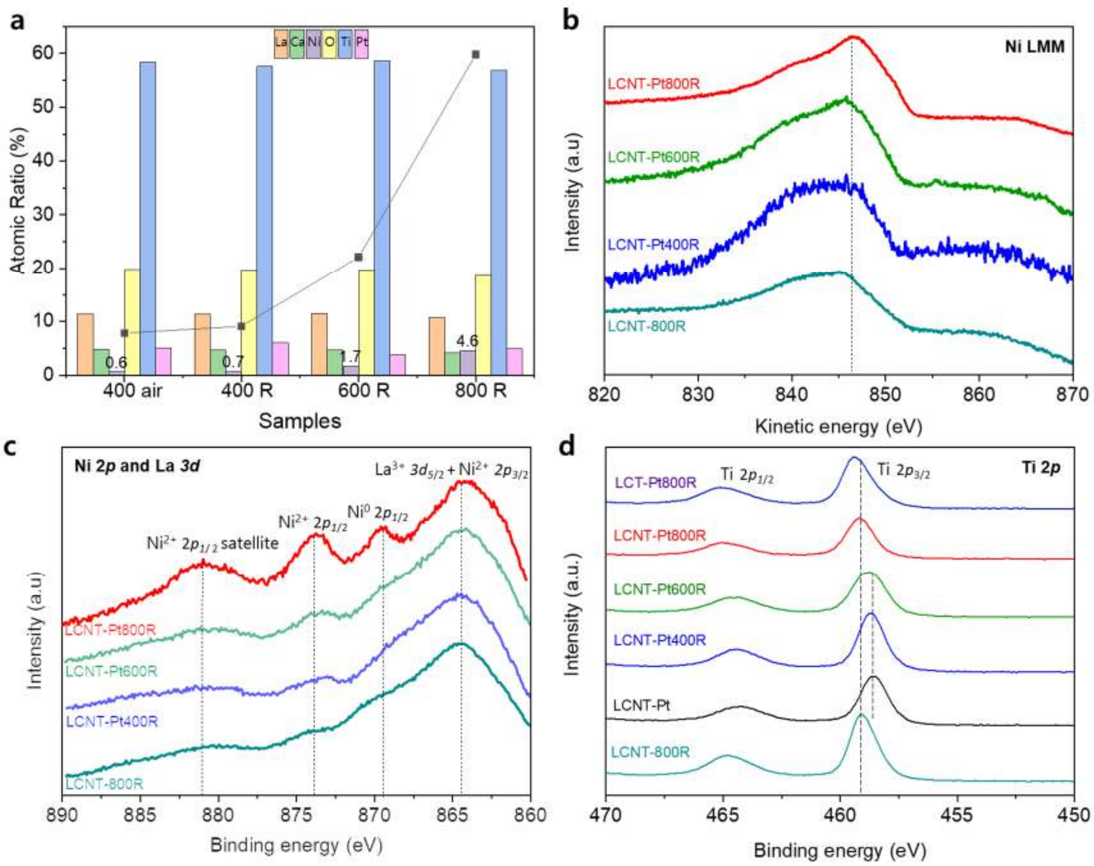

Supplementary Figure 14. XPS analysis for (a) atomic ratios (%) of La, Ca, Ni, Ti, O, Pt species, and XPS spectra of (b) Nickel LMM Auger region, (c) Nickel 2p and La 3d region, (d) Titnium Ti 2p region for calcined samples of LCNT and LCNT-Pt in air, as well as reduced samples of LCNT at 800 ºC, LCT-Pt at 800 ºC and Pt-LCNT at 400 ºC, 600 ºC, 800 ºC for 4 h in 5% H_2/Ar.

A further increase with the existence of Pt was discovered corresponding to the TGA comparisons with oxygen deficiencies of 0.085 and 0.111 for LCNT and LCNT-Pt, respectively. The oxidation state changes indicate the Ni is present in oxide form in LCNT-Pt400R sample while mainly metallic in LCNT-800R and other Pt loaded samples reduced at 600 and 800 ºC, as revealed in the Ni LMM Auger spectra[3] (Fig. S12b). XPS Ti 2p in Fig.S12d also evidenced this behaviour from the peak shift to higher energy. It is worthy to note that the Pt species on the surface may prohibit normal reduction of Ti state in perovskite at exsolution condition[4], removing O rather than Ti that induces the exsolution of cations from electron donation to Pt.

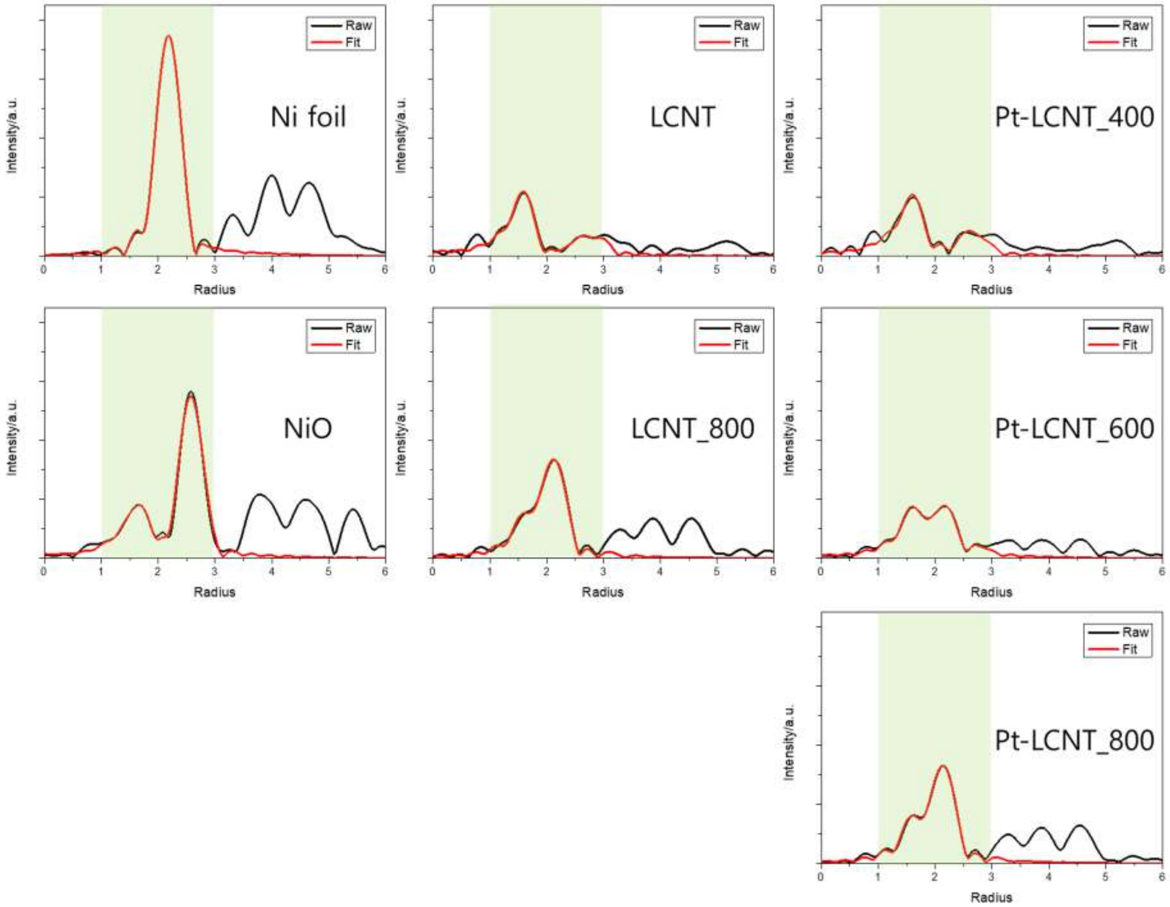

Supplementary Figure 15. EXAFS data analysis of the of Ni K-edge (R-range=1.0–3.0 Å) by comparing experimental data and the fitting curves for the samples of Ni foil, NiO, LCNT, LCNT_800R, LCNT_Pt400R, LCNT_Pt600R, and LCNT_Pt800R.

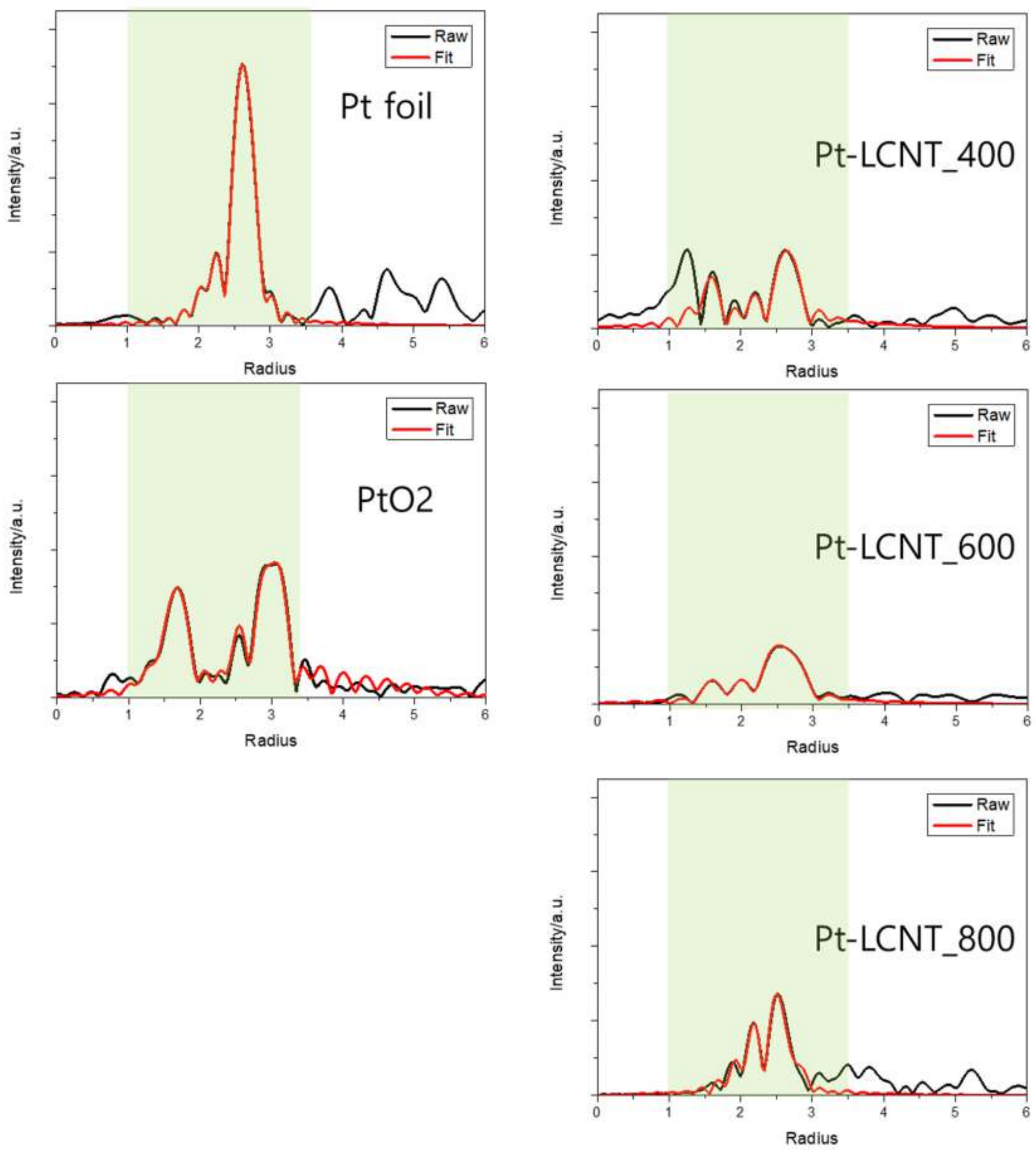

Supplementary Figure 16. EXAFS data analysis of the of Pt L_{III}-edge (R-range=1.0–3.4 Å) by comparing experimental data and the fitting curves for the samples of Pt foil, PtO_2, LCNT_800R, LCNT_Pt400R, LCNT_Pt600R, and LCNT_Pt800R.

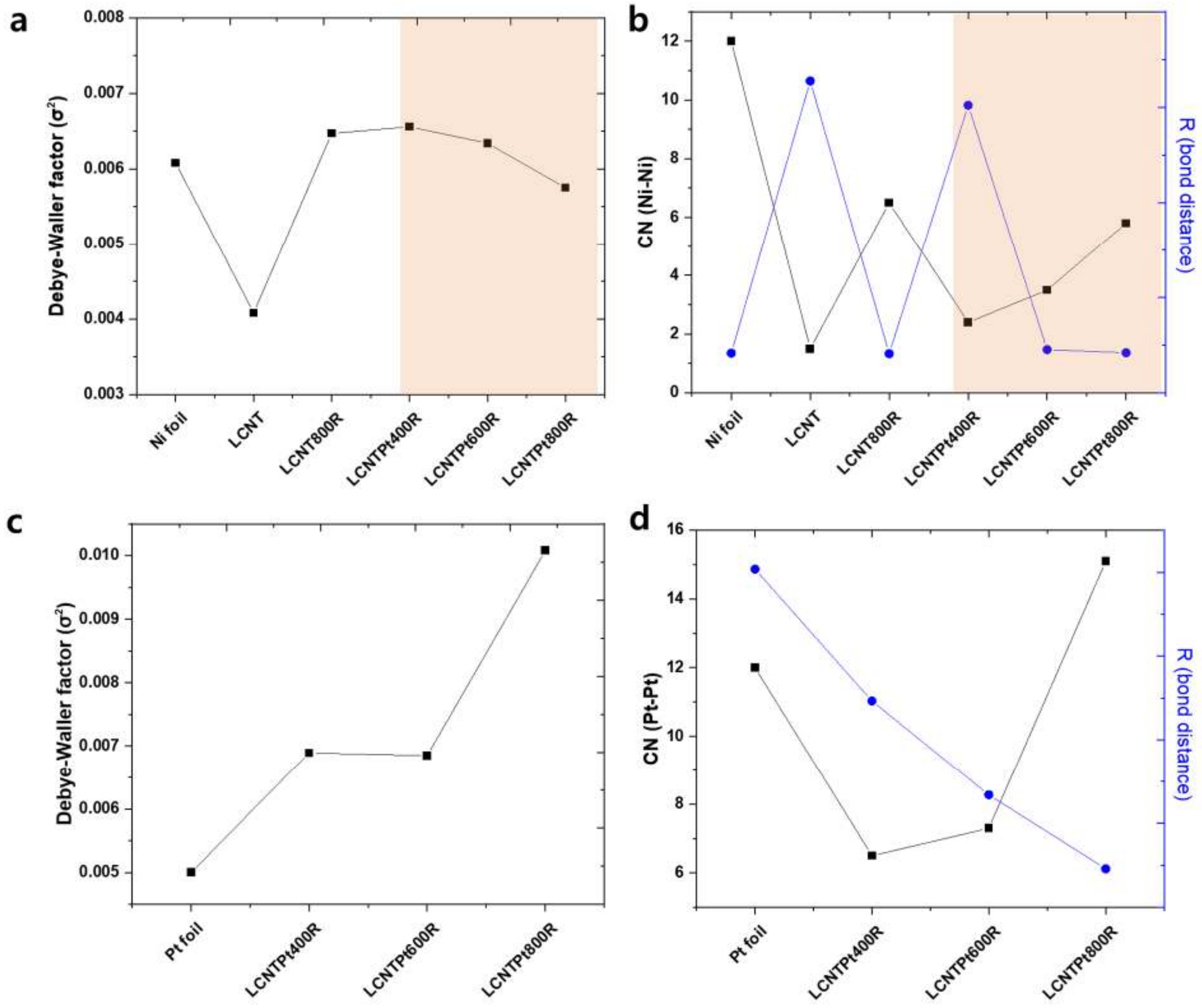

Supplementary Figure 17. Curve-fitting results of Ni K-edge for the change of (a) the Debye-Waller factor, (b) CN (coordination number) and R (bond distance), fitting results of Pt L-edge EXAFS spectra (c) the Debye-Waller factor and (d) CN (coordination number) and R (bond distance).

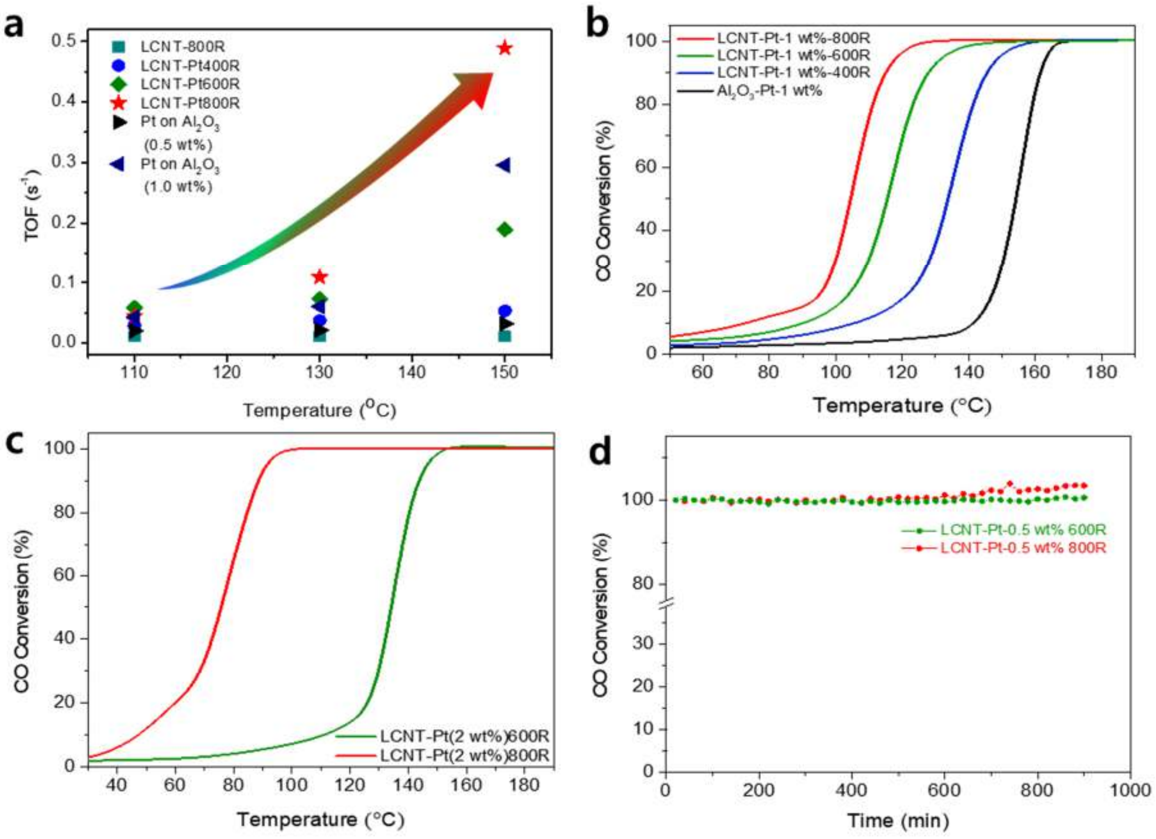

Supplementary Figure 18. CO oxidation performances on (a) calculated TOF_{metal} (s^{-1}) values and light-off curves on the prepared samples with Pt loading of (b) 1 wt.% and (c) 2 wt.% for Pt-LCNT nanofibers reduced at 400 ºC, 600 ºC, 800 ºC by 4 h in 5% H_2/Ar and Pt on Al_2O_3 catalysts as a reference. (d) CO oxidation long-term tests through the catalytic time term method with constant temperatures at T_{99} of 160 ºC and 130 ºC for 0.5 wt% Pt loaded LCNT-600R and -800R, respectively. All experiments were carried out by a feed mixture gas of 20,000 ppm CO, 10.0 vol.% O_2 from air (21% O_2 and 79% N_2) at N_2 balance with a total gas flow rate of 200 ml/min (GHSV=60,000/h).

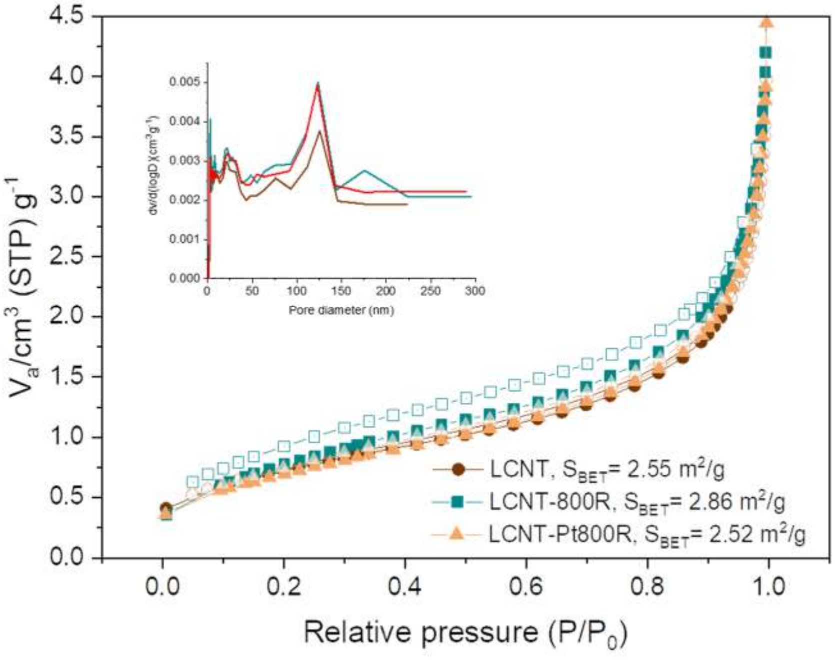

Supplementary Figure 19. Nitrogen adsorption–desorption isotherms of LCNT nanofibers (circle), LCNT reduced nanofibers (square) and LCNT-Pt(0.5wt%) nanofibers after reduced (triangle) at 800 ºC for 4 h in 5% H_2/Ar. (Inset figure: pore size distributions).

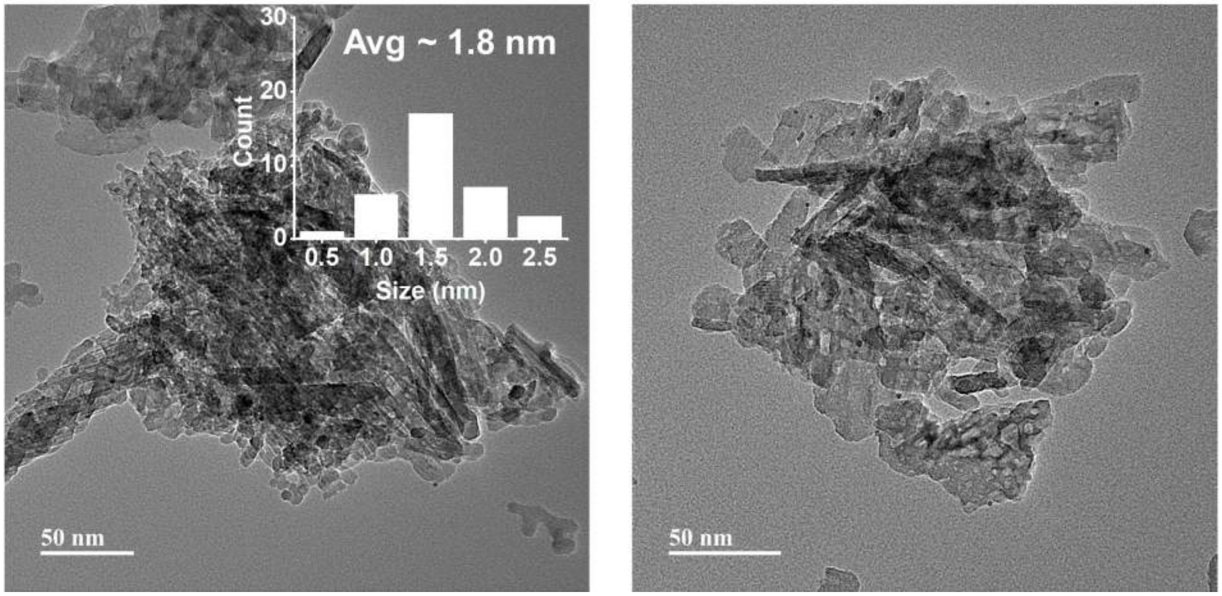

Supplementary Figure 20. TEM images of commercial Pt on γ-Al_2O_3 catalysts (0.5 wt.%, 150 m^2/g) at different sampling region. The embed figure shows the size distribution of Pt nanoparticles.

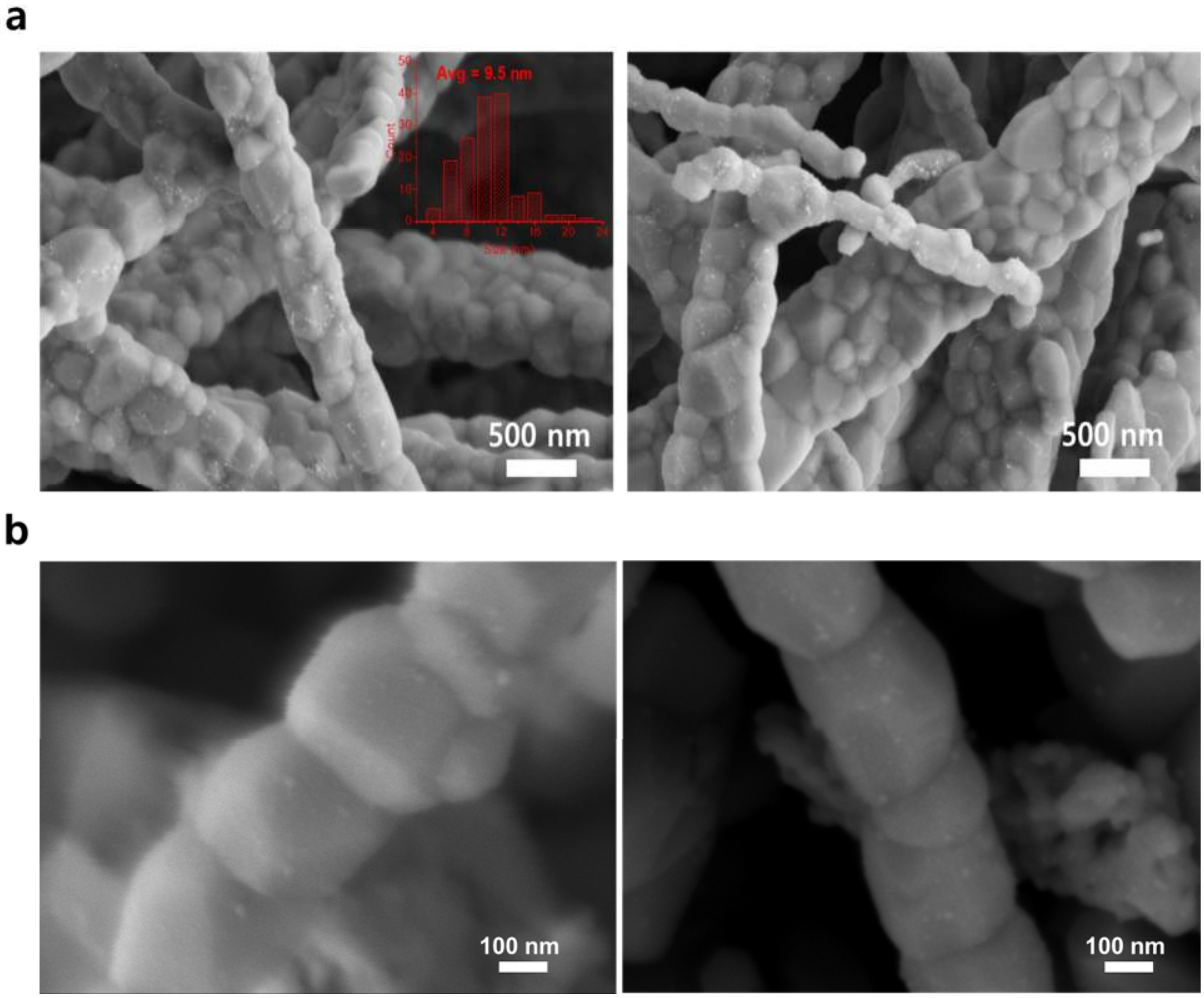

Supplementary Figure 21. SEM images of LCNT-Pt800R (Pt 0.5 wt%) nanofiber catalysts after (a) CO light-off test to 400 °C and (b) long-term CO oxidation test for 40 hours. All experiments were carried out by a feed mixture gas of 20,000 ppm CO, 10.0 vol.% O_2 from air (21% O_2 and 79% N_2) at N_2 balance with a total gas flow rate of 200 ml/min (GHSV=60,000/h).

Supplementary Table 1. Pt content determination by the ICP-OES.

Sample	Blank (ppm)	Test (ppm)	Average (ppm)
LCNT-Pt 0.5 wt%	-0.02	10.40	10.36
	0.01	10.25	
	-0.03	10.43	
LCNT-Pt 0.5 wt%	-0.02	19.20	19.02
	0.01	18.82	
	0.04	19.06	

Supplementary Table 2. The calculated crystallographic parameters from the lattice fringes of HRTEM (Figure 3c). High resolution HAADF of nanoparticle viewed along <110> axis, image corrected for distortion. Particle is ~ 7° away from perovskite zone axis so don't see any lattice fringes from the perovskite, which proves the absence of alignment between metal particles and the support. Because we don't have the perovskite to use as a reference, we need to calibrate the image's pixel size using calculated. Pt d-spacings calculated from AMCSD entry 0011157, Crystal Structures 2nd ed. by Wyckoff.

Direction <hkl>	d [Å]<br>Calculated for <111>	Calculated d [Å]
111 (arrow left)	2.27 ± 0.09	2.27
111 (arrow right)	2.26 ± 0.07	2.27
002	1.94 ± 0.09	1.96
110	1.40 ± 0.07	1.39

Supplementary Table 3. The calculated crystallographic parameters from the lattice fringes of HRTEM (Figure 3d). Here we used the value of 3.87 Å as the value for the perovskite reflections marked in yellow in (d). Nanoparticle <101> and perovskite <100> are parallel. NiPt alloy d-spacings calculated from CIF (ICSD code 646299). a = b = 2.703 Å, c = 3.589 Å, P4/mmm. See excellent agreement between measured & bulk values of d-spacing. Angles between the NiPt planes all agree to within 1° of NiPt if it was Ni_3Pt then a = b = c = 3.750 Å and the angles would all be 60° with equal d-spacings for the [5] planes, i.e. d110 = 1.911 Å. Again the large values of standard deviation arise from variations in d-spacing of the particle which can be seen by simply looking at the FFT in (Figure 3c) the reflections aren't sharp points. Suggests a small degree of disorder within the particle.

hkl	Measured d_{hkl} [Å]	NiPt Alloy d_{hkl} [Å]	Mutual Angle [°]	NiPt Mutual Angle [°]
011	2.161	2.159	(011)-(101) = 70	(011)-(101) = 69
101	2.203	2.159	(101)-(110) = 56	(011)-(110) = 56
110	1.918	1.911	-	-
Perovskite 100	3.870	Value from XRD: 3.870	Perovskite (100) is parallel to NiPt NP (101)	-

Supplementary Table 4. Structural parameters by EXAFS fitting of Ni K-edge (R-range=1.0–3.0 Å) and (b) Pt L_{III}-edge (R-range=1.0–3.4 Å) for the samples of Ni foil, Pt foil, NiO, PtO_2, LCNT, LCNT_800R, LCNT_Pt400R, LCNT_Pt600R, and LCNT_Pt800R. R: bond distance; CN: coordination number; σ^2: Debye-Waller factor. The accuracies of the above parameters were estimated as follows: CN, ±20%; R, ±1%; σ^2, ±20%. The data ranges used for data fitting in R space (ΔR) is 1.2−3.0 Å, respectively.

Samples	Bonding	S_0^2	CN	σ^2	*R* (Å)	delr	ΔE0 (eV)	R-factor
Ni foil	Ni-Ni1.1	0.784 (+/- 0.02785)	12	0.00608 (+/- 0.00061)	2.48299	0.00399 (+/- 0.00228)	6.435 (+/- 0.39514)	0.001836
NiO	Ni-O	1.091 (+/- 0.14551)	6	0.00712 (+/- 0.00205)	2.08971	-0.01859 (+/- 0.01448)	-1.126 (+/- 1.58445)	0.004795
	Ni-Ni3.1	1.150 (+/- 0.10931)	12	0.00747 (+/- 0.00077)	2.94832	-0.03328 (+/- 0.00654)	-3.739 (+/- 0.94334)	
PtO_2	Pt-O4.1	1.346 (+/- 0.13838)	4	0.00300 (+/- 0.00086)	2.02415	0.08265 (+/- 0.00843)	11.996 (+/- 1.36978)	0.017799
Pt foil	Pt-Pt1.1	0.888 (+/- 0.03896)	12	0.00500 (+/- 0.00013)	2.76073	-0.01337 (+/- 0.00156)	7.457 (+/- 0.56982)	0.002623
LCNT	Ni-Ni1.1	0.099 (+/- 0.34450)	1.5	0.00408 (+/- 0.01941)	3.05513	0.07353 (+/- 0.14588)	18.163 (+/- 3.19012)	0.002419
	Ni-O	1.010 (+/- 0.21280)	5.6	0.00558 (+/- 0.00281)	2.02375	-0.08455 (+/- 0.02196)	-7.072 (+/- 3.21142)	
	Ni-Ni3.1	0.620 (+/- 3.83117)	6.5	0.04156 (+/- 0.12162)	2.69102	0.21202 (+/- 0.42924)	15.849 (+/- 1.17392)	
LCNT_800R	Ni-Ni1.1	0.426 (+/- 0.34202)	6.5	0.00647 (+/- 0.00739)	2.48203	0.00303 (+/- 0.06027)	-8.296 (+/- 1.87278)	0.001933
	Ni-O	0.585 (+/- 0.14221)	3.2	0.00611 (+/- 0.00261)	2.01266	-0.09564 (+/- 0.02512)	-8.140 (+/- 2.09374)	
	Ni-Ni3.1	0.480 (+/- 5.09909)	5.0	0.03324 (+/- 0.16022)	3.26608	0.28448 (+/- 0.84615)	13.342 (+/- 4.37102)	
LCNT_Pt400R	Ni-Ni1.1	0.160 (+/- 0.37990)	2.4	0.00656 (+/- 0.01818)	3.00423	0.02263 (+/- 0.15753)	7.438 (+/- 2.06733)	0.010293
	Ni-O	1.009 (+/- 1.77218)	5.5	0.00565 (+/- 0.01186)	2.04872	-0.05958 (+/- 0.11107)	-1.057 (+/- 1.65242)	

Continuous to Table S4								
	Ni-Ni3.1	0.597 (+/- 0.07768)	6.2	0.07420 (+/- 0.12867)	2.30268	-0.17632 (+/- 0.53037)	-16.400 (+/- 3.35064)	
	Pt-O4.1	0.539 (+/- 0.13582)	1.6	0.00314 (+/- 0.00194)	1.97203	0.03053 (+/- 0.02071)	-6.870 (+/- 4.54990)	0.015951
	Pt-Pt1.1	0.481 (+/- 0.05831)	6.5	0.00689 (+/- 0.00079)	2.72933	-0.04477 (+/- 0.00704)	5.542 (+/- 1.13133)	
LCNT_Pt600R	Ni-Ni1.1	0.231 (+/- 0.18755)	3.5	0.00634 (+/- 0.00595)	2.49052	0.01151 (+/- 0.04445)	-5.888 (+/- 7.75889)	0.001373
	Ni-O	0.734 (+/- 0.27440)	4.0	0.00552 (+/- 0.00465)	2.02114	-0.08716 (+/- 0.03312)	-6.941 (+/- 4.12187)	
	Ni-Ni3.1	0.381 (+/- 0.13763)	4.0	0.00219 (+/- 0.02141)	3.00190	0.02030 (+/- 0.19481)	2.896 (+/- 1.80672)	
	Pt-O4.1	0.469 (+/- 0.16118)	1.4	0.00349 (+/- 0.00377)	2.03707	0.09557 (+/- 0.02656)	19.678 (+/- 2.30275)	0.002511
	Pt-Pt1.1	0.540 (+/- 0.06501)	7.3	0.00685 (+/- 0.00067)	2.70677	-0.06733 (+/- 0.00649)	2.182 (+/- 1.48759)	
LCNT_Pt800R	Ni-Ni1.1	0.377 (+/- 0.14427)	5.8	0.00575 (+/- 0.00268)	2.48383	0.00483 (+/- 0.02792)	-8.124 (+/- 2.90573)	0.001571
	Ni-O	0.503 (+/- 0.27997)	2.8	0.00431 (+/- 0.00617)	2.00399	-0.10431 (+/- 0.04661)	-8.568 (+/- 1.90451)	
	Ni-Ni3.1	0.227 (+/- 2.48041)	2.4	0.02525 (+/- 0.14915)	3.16324	0.18164 (+/- 0.73027)	5.955 (+/- 1.87823)	
	Pt-O4.1	0.124 (+/- 0.09495)	0.4	-0.00269 (+/- 0.00161)	2.49468	0.55318 (+/- 0.02172)	-10.214 (+/- 1.76314)	0.021005
	Pt-Pt1.1	1.121 (+/- 0.19882)	15.1	0.01008 (+/- 0.00105)	2.68918	-0.08492 (+/- 0.01143)	1.972 (+/- 0.49757)	

Supplementary Table 5. Summarized table for binding energies (eV) and relative percentages for each oxygen species on the surface of samples. (O_1: lattice oxygen, O_2: defects or surface oxygen)[6].

	**LCNT-800R**	**LCNT-Pt (oxidised)**	**LCNT-Pt 400R**	**LCNT-Pt 600R**	**LCNT-Pt 800R**	**LCT-Pt 800R**
O1-eV	530.32	529.81	530.32	530.03	530.39	530.53
%	90.33	60.13	90.37	72.81	59.08	84.07
O2-eV	532.23	530.84	532.23	531.53	530.57	532.63
%	9.67	39.87	9.63	27.19	40.92	15.93

Supplementary References:

1 Xue, J., Wu, T., Dai, Y. & Xia, Y. Electrospinning and electrospun nanofibers: methods, materials, and applications. *Chem. Rev.* **119**, 5298-5415, (2019).

2 Henry, C. R. Morphology of supported nanoparticles. *Prog. Surf. Sci.* **80**, 92-116, (2005).

3 Biesinger, M. C., Lau, L. W., Gerson, A. R. & Smart, R. S. The role of the Auger parameter in XPS studies of nickel metal, halides and oxides. *Phys Chem Chem Phys* **14**, 2434-2442, (2012).

4 Neagu, D. *et al.* Nano-socketed nickel particles with enhanced coking resistance grown in situ by redox exsolution. *Nat. Commun.* **6**, 8120, (2015).

5 Ferromagnetism in $LaCoO_3$. *PHYSICAL REVIEW B.* **70**, (2004).

6 Kothari, M. *et al.* Platinum incorporation into titanate perovskites to deliver emergent active and stable platinum nanoparticles. *Nat. Chem.* **13**, 677–682, (2021).
